# Analysis of the entire mitochondrial genome reveals Leber’s hereditary optic neuropathy mitochondrial DNA mutations in an Arab cohort with multiple sclerosis

**DOI:** 10.1038/s41598-022-15385-2

**Published:** 2022-06-30

**Authors:** Ghada Al‐Kafaji, Maram A. Alharbi, Hasan Alkandari, Abdel Halim Salem, Moiz Bakhiet

**Affiliations:** 1 Department of Molecular Medicine and Al-Jawhara Centre for Molecular Medicine, Genetics, and Inherited Disorders, College of Medicine and Medical Sciences, Arabian Gulf University, Manama, Kingdom of Bahrain; 2 College of Forensic Sciences, Naif Arab University for Security Sciences, Riyadh, Kingdom of Saudi Arabia; 3 Department of Anatomy, College of Medicine and Medical Sciences, Arabian Gulf University, Manama, Kingdom of Bahrain; 4 Department of molecular Medicine and Al-Jawhara Centre for Molecular Medicine, Genetics and Inherited Disorders, College of Medicine and Medical Sciences, Arabian Gulf University, Salmaniya Avenue, Building 293, Road 2904, Block 329, Manama, Kingdom of Bahrain

**Keywords:** Genetics, Neuroscience

## Abstract

Several mitochondrial DNA (mtDNA) mutations of Leber's hereditary optic neuropathy (LHON) have been reported in patients with multiple sclerosis (MS) from different ethnicities. To further study the involvement of LHON mtDNA mutations in MS in the Arab population, we analyzed sequencing data of the entire mitochondrial genome from 47 unrelated Saudi individuals, 23 patients with relapse-remitting MS (RRMS) and 24 healthy controls. Ten LHON mutations/variants were detected in the patients but were absent in the controls. Of them, the common primary pathogenic mutation m.14484T>C and the rare mutation m.10237T>C were found in one patient, whereas the rare mutation m.9101T>C was found in another patient. The remaining were secondary single nucleotide variants (SNVs) found either in synergy with the primary/rare mutations or individually in other patients. Patients carrying LHON variants also exhibited distinct mtDNA variants throughout the mitochondrial genome, eight were previously reported in patients with LHON. Moreover, five other LHON-related SNVs differed significantly in their prevalence among patients and controls (P < 0.05). This study, the first to investigate LHON mtDNA mutations/variants in a Saudi cohort may suggest a role of these mutations/variants in the pathogenesis or genetic predisposition to MS, a possibility which needs to be explored further in a large-scale.

## Introduction

Multiple sclerosis (MS) is a progressive neurological disease characterized by autoimmune inflammation coupled with demyelination and neurodegeneration^1,2^. The disease which affects over two million people worldwide, is more common in females than in males^2,3^. The exact cause of MS is still unclear, but it is believed that genetic predisposition, epigenetic factors and various environmental factors such as infections, vitamin D deficiency, and smoking contribute significantly to the development of the disease^4,5^. These agents are able to trigger a cascade of events in the immune system including an accumulation of macrophages in microglia in the brain and lymphocytes in the white matter and the gray matter of the central nervous system (CNS), leading to demyelination and destruction of axons^6^.

In recent years, mitochondrial dysfunction has been shown to occur early in MS and plays an important role in the axonal degeneration and demyelination^7–9^. Abnormalities in the mitochondrial genome including defects in the mitochondrial DNA (mtDNA), altered mtDNA content and dysregulation of mitochondrial gene expression, which ultimately lead to increase production of free radical and oxidative damage, have all been reported in patients with MS^9,10^. The main role of mitochondria is to produce energy through oxidative phosphorylation (OXPHOS). The human mtDNA is a 16.6 kb circular double-stranded DNA molecule which encodes 13 OXPHOS proteins, 22 tRNA genes and 2 rRNA genes. The mtDNA-encoded genes include seven subunits of NADH dehydrogenase of complex I (ND 1, 2, 3, 4L, 4, 5 and 6); cytochrome b-c1 (CYB) of ubiquinol-cytochrome c reductase of complex III; three subunits of cytochrome c oxidase of complex IV (COI, II, III); and two subunits of mitochondrial ATP synthase of complex V (ATP6 and ATP8). Due to the multi-copy nature of the mitochondrial genome, some mutations affect all copies of the mtDNA (homoplasmic), whereas others only present in some copies of the mtDNA (heteroplasmic)^11^.

Multiple lines of evidence have suggested that mtDNA could play a role in MS. In this context, mutations and common polymorphisms in mtDNA have been reported in MS patients. For instance, variants in the *ND2* gene and other mtDNA-encoded genes have been implicated in the susceptibility to MS^12,13^. Moreover, a previous study by our group identified four novel mutations in the mtDNA-encoded *ND4* gene in patients with MS, which caused complex I dysfunction and could be implicated in the pathogenicity of MS^14^. Though the involvement of mtDNA in the pathogenesis of MS was initially proposed based on the observation of mother-to-child transmission in familial cases^15,16^, and the coexistence of symptoms of inflammatory demyelination similar to MS in Leber’s hereditary optic neuropathy (LHON), a maternally inherited mitochondrial blinding disorder caused by mtDNA mutations^17^.

One of three primary mutations m.3460G>A, m.11778G>A and m.14484T>C respectively affecting the *MT-ND1*, *MT-ND4* and *MT-ND6* subunit genes of NADH dehydrogenase (complex I) are present in more than 90% of all LHON cases^18,19^. Other mtDNA mutations that cause LHON appear to be relatively rare within the population and have been found in singleton cases or in a single family, and their strict pathogenicity awaiting further confirmation^18,19^. It has been shown that only about 50% of males and 10% of females who harbour pathogenic LHON mutations develop optic neuropathy^20,21^. This incomplete penetrance and gender bias of males being more affected than females imply that additional mitochondrial and nuclear genetic factors or environmental factors contribute to the phenotypic expression and severity of the disease^18–21^. On the other hand, a number of mtDNA variants known as “secondary mutations” have been shown to influence the penetrance and clinical expression of these primary mutations^22–24^. Secondary mutations have been also found at higher frequency in LHON patients than in controls and thus may play an additional role in the pathogenesis/risk of the disease^22–24^. In an early study to explore a possible connection between MS and LHON, Harding et al.^17^ described the primary mutation m.11778G>A in eight LHON women who developed neurological features compatible with a diagnosis of MS. This was followed by many other reports which verified the presence of primary LHON mutations in a sub-group of MS patients or patients with MS-like syndrome in the European and North American populations^25–28^. While a study by Vanopdenbosch et al.^29^ showed that the association of LHON primary mutations with MS is more than a coincidence and that carrying a primary mutation is a risk factor for developing MS, at least in Caucasian populations. In contrast, none of the most common LHON primary mutations were detected in Japanese or Korean patients with MS^30,31^. Additionally, certain sets of secondary LHON-related mDNA variants were found to contribute to the genetic susceptibility of MS in specific ethnic groups^32,33^. These observations suggest that the presence and relative frequencies of LHON variants vary among individuals of different ethnicities, possibly due to genetic, racial and other factors, which differ in different populations. Aside from these studies, no information is available on the incidence of LHON variants in MS patients in the Arab population. We recently sequenced the entire mitochondrial genome from unrelated Saudi Arab individuals including 23 patients with relapse-remitting MS (RRMS) and 24 healthy controls using next-generation sequencing (NGS), and we found several unique and common mtDNA mutations/variants^34^. In this study we analyzed the generated large-scale genomic sequence data of the entire mtDNA to investigate the presence of variants involved in or associated with LHON in this Saudi cohort. Knowledge about LHON-associated mtDNA variants can assist in the understanding of genetic contribution of LHON to the pathogenesis and genetic predisposition to MS.

## Results

### Characteristics of study subjects

The characteristics of 47 Saudi subjects including 23 patients diagnosed with RRMS according to McDonald diagnosis criteria^35^, and 24 healthy controls are illustrated in Table 1. The data are presented as number or mean ± standard deviation (SD). The patient group consisted of 5 males and 18 females. The mean age of the patient group was 28 ± 7.5 within a range between 18 and 44 years. The control group consisted of 5 males and 19 females. The mean age of the control group was 31 ± 7.5 within a range between 22 and 52 years. There were no significant differences between patients and controls in the mean age, sex distribution, mean BMI and mean blood pressure (P > 0.05). For patient group, the mean disease duration was 5.3 years ± 4 and ranged from 1 to 15 years, the mean EDSS was 3.9 ± 1.4 and ranged from 2 to 6.5. Most of the patients presented with numbness and visual problem (23 and 20 respectively). Eighteen patients had muscle spasticity, 13 patients had balance problem, 10 patients suffered from slurred speech and 14 patients suffered from a psychiatric condition, most commonly depression. The patients were under the following treatment: Avonex (n = 3), Betaferon (n = 8), Glienya (n = 3), Rebif (n = 5) and Tysabri (n = 4).

**Table 1:** Demographic and clinical data of RRMS patients and healthy controls.

	Patients (n = 23)	Controls (n = 24)
Age (mean ± SD)	28 ± 7.5	31 ± 7.5
Gender ratio (male/female)	5/18	5/19
BMI (mean ± SD)	27 ± 6.2	29 ± 5.5
**Blood pressure (mean ± SD)**		
Systolic	123 ± 11.4	131 ± 16.4
Diastolic	72 ± 11.9	76 ± 9.6
Disease duration (mean ± SD)/range	5.3 ± 4.0/(1–15)	
EDSS (mean ± SD)/range	3.9 ± 1.4/(2–6.5)	
**Medication**		
Avonex	3	
Betaferon	8	
Glienya	3	
Rebif	5	
Tysabri	4	

*RRMS* relapse-remitting multiple sclerosis, *EDSS* Expanded Disability Status Scale. Data are presented as number or mean ± standard deviation (SD).

### Analysis of mtDNA sequences in RRMS patients and healthy controls

We analyzed our recently generated large-scale genomic sequence data of the entire mitochondrial genome from 23 patients with RRMS and 24 healthy controls^34^. The mtDNA sequences from the patients and controls were compared to the revised rCRS and nucleotide variants recorded, and all mtDNA variants were submitted to the GenBank database^34^.

### Identification of LHON mtDNA mutations/variants in RRMS patients

**Figure 1:**
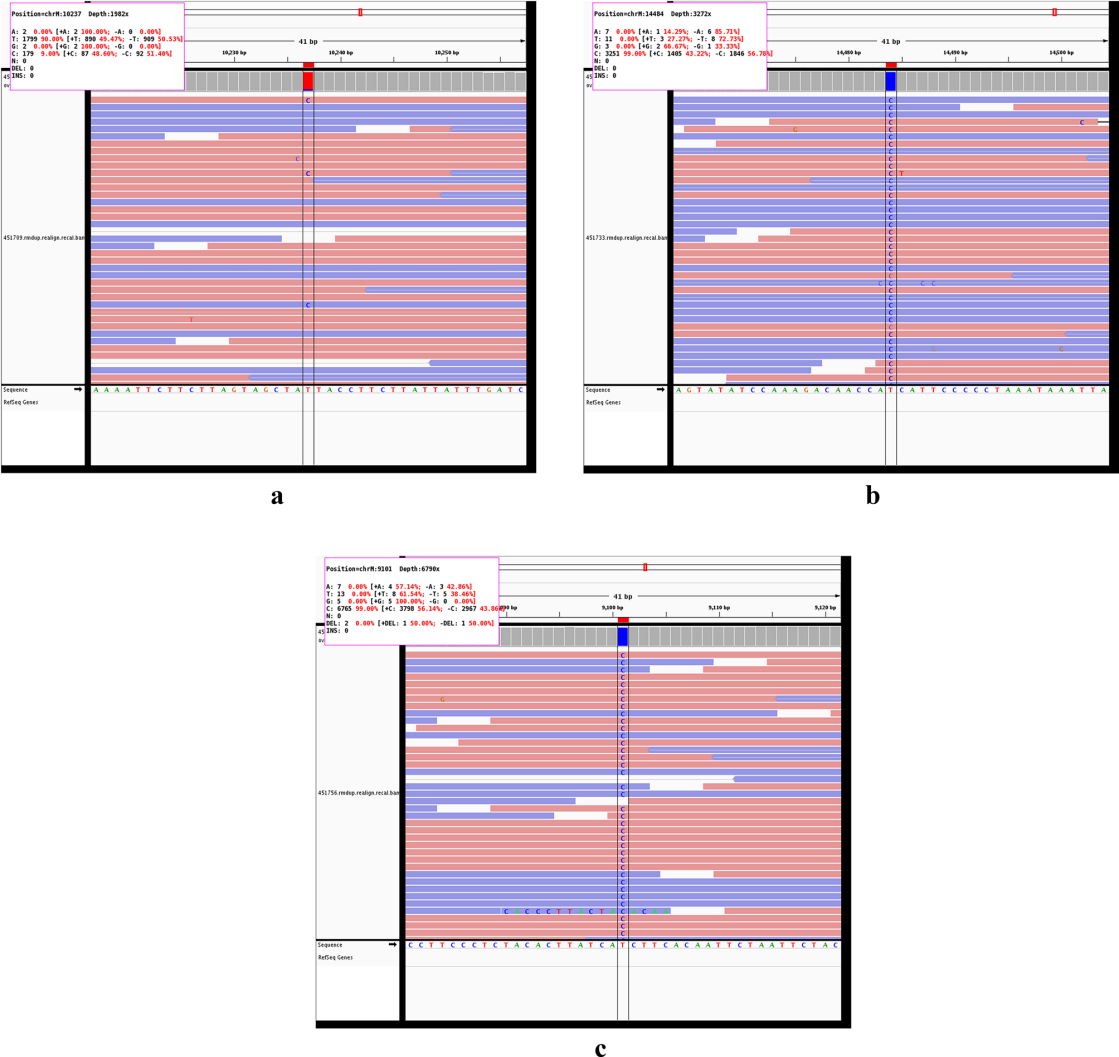
HiSeq X NGS short reads of rare and primary LHON mutations found in two RRMS patients. (**a**) m.10237T>C (p.Ile60Thr) rare mutation of *MT-ND3* gene. (**b**) m.14484T>C (p.Met64Val) primary mutation of *MT-ND6* gene. (**c**) m.9101T>C (p.Ile192Thr) rare mutation of *MT-ATP6* gene.

mtDNA variants were annotated using the MITOMAP database system for the human mitochondrial genome (http://www.mitomap.org/MITOMAP) and other databases, in which mtDNA variants are classified into three categories: Benign variants, unclassified variants or mutations^36^. Analysis of the entire mtDNA sequences in RRMS patients revealed ten single nucleotide variants (SNVs) described as missense mutations in protein-coding genes, which were detected in six patients. Details of these SNVs are shown in Table 2. All of the identified SNVs were previously reported in the MTOMAP and other databases to be associated with LHON. Of them, two pathogenic mutations were detected in one patient (P2) namely the primary homoplasmic mutation m.14484T>C in *MT-ND6* gene and the rare heteroplasmic mutation m.10237T>C in *MT-ND3* gene. Whereas one rare homoplasmic mutation namely m.9101T>C in *MT-ATP6* gene was found in another patient (P15). Short reads mapped against mitochondrial genome of these mutations are shown in Fig. 1.

**Table 2:** LHON mtDNA mutations identified in RRMS patients and their deleteriousness prediction.

Patient ID	Gene	Nucleotide change	Amino acid change	Type of mutation	Homoplasmy/heteroplasmy	Category of LHON*	Bioinformatics tools			
							PolyPhen	SIFT	CAAD	Mutation assessor
							Prediction/score	Prediction/score	Prediction/score	Prediction/score
P1	*MT-ND2*	m.4695T>C	p.Phe76Leu	Missense	Homoplasmy	Secondary	Benign/0	Tolerated/0.84	Neutral/0.23	Low/− 0.78
P2	*MT-ND2*	m.5442T>C	p.Phe325Leu	Missense	Homoplasmy	Secondary	Benign/	Tolerated/0.96	Neutral/− 0.1	Low/− 1.74
	***MT-ND3***	**m.10237T>C**	**p.Ile60Thr**	**Missense**	**Heteroplasmy**	**Rare**	**Probably damaging/0.96**	**Damaging/0.09**	**Damaging/3.33**	**High/3.86**
	*MT-ND5*	m.13105A>G	p.Ile257Val	Missense	Homoplasmy	Secondary	Benign/0.01	Tolerated/0.52	Neutral/− 0.58	Low/− 0.72
	***MT-ND6***	**m.14484T>C**	**p.Met64Val**	**Missense**	**Homoplasmy**	**Primary**	**Probably damaging/0.99**	**Damaging/0.19**	**Damaging/0.89**	**High/3.06**
P15	***MT-ATP6***	**m.9101T>C**	**p.Ile192Thr**	**Missense**	**Homoplasmy**	**Rare**	**Benign/0.01**	**Tolerated/1**	**Neutral/− 1.09**	**Low/− 1.17**
	*MT-ND5*	m.12358A>G	p.Thr8Ala	Missense	Homoplasmy	Secondary	Benign/0	Tolerated/0.47	Neutral/0.34	Medium/1.04
P16	*MT-ND2*	m.4917A>G	p.Asn150Asp	Missense	Homoplasmy	Secondary	Benign/0.06	Tolerated/0.22	Neutral/0.7	Moderate/1.4
P18	*MT-ND1*	m.3316G>A	p.Ala4Thr	Missense	Homoplasmy	Secondary	Benign/0	Tolerated/0.48	Neutral/1.04	Low/− 0.76
P22	*MT-ND1*	m.3533C>T	p.Thr76Ile	Missense	Homoplasmy		Benign/0.02	Tolerated/0.56	Neutral/0.12	Low/0.12

*LHON* Leber’s hereditary optic neuropathy, *RRMS* relapse-remitting multiple sclerosis, *EDSS* Expanded Disability Status Scale. *LHON Mutations were reported as primary or rare (bold) and secondary according to MITOMAP, HmtDB, ClinVar and MEDLINE-listed publications on life sciences. *PolyPhen* Polymorphism Phenotyping, *SIFT* Sorting Intolerant From Tolerant, *CADD* Combined Annotation Dependent Depletion.

Seven other secondary variants, all in the homoplasmic state, were found either in synergy with the primary/rare mutations or individually in other patients. These include m.5442T>C in *MT-ND2* gene and m.13105A>G in *MT-ND5* gene, both were detected in patient P2; m.12358A>G in *MT-ND5* gene was detected in patient P15. The remaining variants m.4695T>C in *MT-ND2* gene, m.4917A>G in *MT-ND2* gene, m.3316G>A in *MT-ND1* gene and m.3533C>T in *MT-ND1* were found in four other patients (P1, P16, P18 and P22) respectively. Bioinformatics analysis of the deleteriousness prediction of these variants using the PolyPhen2, SIFT, CADD, and Mutation Assessor models^37^ confirmed the pathogenicity of m.14484T>C and m.10237T>C mutations which were detected in patient P2, while all other variants were predicted as benign (Table 2).

### Characteristics of RRMS patients carrying LHON mtDNA mutations/variants

Table 3 shows the characteristics of RRMS patients who harboured one of the LHON mutations/variants in mtDNA-encoding genes. Patient P2 was a 20 year-old male with a disease duration of 1 years and an EDSS of 3. He carried four variants; the primary mutation m.14484T>C in *MT-ND6 gene*, the rare mutation m.10237T>C in *MT-ND3* gene, and the secondary variants m.5442T>C in *MT-ND2* gene and m.13105A>G in *MT-ND5* gene. Whereas patient P15 was a 27 year-old female with a disease duration of 3 years and an EDSS of 3. She carried the rare mutation m.9101T>C in *MT-ATP6* gene and the secondary variant m.12358A>G in *MT-ND5* gene. All other patients who carried secondary LHON-related variants were young adult females aged from 20 to 34 years old and had disease durations from 1 to 5 years and EDSS scales from 2.5 to 3.5. All of the patients were reported with numbness and visual problem along with other neurological dysfunctions.

**Table 3:** Characteristics of RRMS patients carrying LHON mtDNA mutations.

Patient ID	Gender	Age (years)	Disease duration (years)	EDSS	Main neurological dysfunction	Gene	Nucleotide change	Amino acid change	Type of mutation	Homoplasmy/heteroplasmy	Category of LHON*
P1	Female	24	5	3.5	Numbness, visual problem muscle weakness, balance problem	*MT-ND2*	m.4695T>C	p.Phe76Leu	Missense	Homoplasmy	Secondary
P2	Male	20	1	3	Numbness, visual problem muscle weakness	*MT-ND2*	m.5442T>C	p.Phe325Leu	Missense	Homoplasmy	Secondary
						***MT-ND3***	**m.10237T>C**	**p.Ile60Thr**	**Missense**	**Heteroplasmy**	**Rare**
						*MT-ND5*	m.13105A>G	p.Ile257Val	Missense	Homoplasmy	Secondary
						***MT-ND6***	**m.14484T>C**	**p.Met64Val**	**Missense**	**Homoplasmy**	**Primary**
P15	Female	27	3	3	Numbness, visual problem muscle weakness	***MT-ATP6***	**m.9101T>C**	**p.Ile192Thr**	**Missense**	**Homoplasmy**	**Rare**
						*MT-ND5*	m.12358A>G	p.Thr8Ala	Missense	Homoplasmy	Secondary
P16	Female	21	2	3	Numbness, visual problem muscle weakness	*MT-ND2*	m.4917A>G	p.Asn150Asp	Missense	Homoplasmy	Secondary
P18	Female	34	3	3.5	Numbness, visual problem muscle weakness, balance problem	*MT-ND1*	m.3316G>A	p.Ala4Thr	Missense	Homoplasmy	Secondary
P22	Female	21	3	2.5	Numbness, visual problem muscle weakness	*MT-ND1*	m.3533C>T	p.Thr76Ile	Missense	Homoplasmy	Secondary

*RRMS* relapse-remitting multiple sclerosis, *LHON* Leber’s hereditary optic neuropathy, *EDSS* Expanded Disability Status Scale. *LHON Mutations were reported as primary or rare (bold) and secondary according to MITOMAP, HmtDB, ClinVar and MEDLINE-listed publications on life sciences.

### Other variants in RRMS patients carrying LHON mtDNA mutations

We next investigated the presence of other variants in the six RRMS patients who carried one of the LHON mtDNA mutations. As shown in Table 4, the patients exhibited distinct SNVs in different regions of the mitochondrial genome. Of these, eight were previously reported in patients with LHON. Two variants namely the heteroplasmic m.189A>G and the homoplasmic m.236T>C in the D-loop region were found in patient P2. This patient also carried four homoplasmic silent variants namely m.10810T>C, m.10915T>C, m.11176G>A, and m.12007G>A in *MT-ND4* gene. Additional homoplasmic silent variant in *MT-ND4* gene namely m.10876A>G was found in another patient (P15). Finally, one homoplasmic variant namely m.15928G>A in *MT-TT* tRNA gene was found in patient P16.

**Table 4:** Other variants in RRMS patients carrying one of the LHON mtDNA mutations.

Patient ID	Locus	Nucleotide change	Amino acid change	Type of variant	Homoplasmy/heteroplasmy	Patient ID	Locus	Nucleotide change	Amino acid change	Type of variant	Homoplasmy/heteroplasmy
P1	MT-RNR2	m.1812C>T	–	Substitution	Homoplasmy	P15	D-loop	m.217T>C	–	Substitution	Homoplasmy
P2	D-loop	m.58T>C	–	Substitution	Heteroplasmy		D-loop	m.340C>T	–	Substitution	Homoplasmy
	**D-loop**	**m.189A>G**	–	**Substitution**	**Heteroplasmy**		D-loop	m.16234C>T	–	Substitution	Homoplasmy
	**D-loop**	**m.236T>C**	–	**Substitution**	**Homoplasmy**		D-loop	m.16257C>T	–	Substitution	Homoplasmy
	D-loop	m.247G>A	–	Substitution	Homoplasmy		D-loop	m.16259C>T	–	Substitution	Homoplasmy
	D-loop	m.16114C>A	–	Substitution	Homoplasmy		D-loop	m.16269A>G	–	Substitution	Homoplasmy
	D-loop	m.16148C>T	–	Substitution	Homoplasmy		D-loop	m.16290C>T	–	Substitution	Homoplasmy
	D-loop	m.16168C>T	–	Substitution	Homoplasmy		*MT-RNR1*	m.827A>G	–	Substitution	Heteroplasmy
	D-loop	m.16186C>T	–	Substitution	Homoplasmy		*MT-TT*	m.15907A>G	–	Substitution	Homoplasmy
	D-loop	m.16230A>G	–	Substitution	Homoplasmy		*MT-ND1*	m.3720A>G	p.Gln138(=)	Silent	Homoplasmy
	D-loop	m.16293A>G	–	Substitution	Homoplasmy		*MT-ND2*	m.5390A>G	p.Met307(=)	Silent	Homoplasmy
	*MT-RNR1*	m.825T>A	–	Substitution	Homoplasmy		*MT-ND2*	m.5426T>C	p.His319(=)	Silent	Homoplasmy
	*MT-RNR1*	m.1048C>T	–	Substitution	Homoplasmy		*MT-CO1*	m.6045C>T	p.Leu48(=)	Silent	Homoplasmy
	*MT-RNR2*	m.2245A>G	–	Substitution	Homoplasmy		*MT-CO1*	m.6152T>C	p.Val83(=)	Silent	Homoplasmy
	*MT-RNR2*	m.2885T>C	–	Substitution	Heteroplasmy		*MT-CO2*	m.8155G>A	p.Gly190(=)	Silent	Heteroplasmy
	*MT-TI*	m.4312C>T	–	Substitution	Homoplasmy		***MT-ND4***	**m.10876A>G**	**p.Leu39(=)**	**Silent**	**Homoplasmy**
	*MT-TA*	m.5603C>T	–	Substitution	Homoplasmy		*MT-ND5*	m.13020T>C	p.Gly228(=)	Silent	Homoplasmy
	*MT-ND1*	m.3516C>A	p.Leu70(=)	Silent	Homoplasmy		*MT-ND5*	m.13734T>C	p.Phe466(=)	Silent	Homoplasmy
	*MT-ND2*	m.4586T>C	p.Ala39(=)	Silent	Homoplasmy	P16	*MT-RNR2*	m.1888G>A	–	Substitution	Homoplasmy
	*MT-ND2*	m.5073A>G	p.Ile202Val	Missense	Homoplasmy		*MT-TR*	m.10463T>C	–	Substitution	Homoplasmy
	*MT-ND2*	m.5096T>C	p.Ile209(=)	Silent	Homoplasmy		***MT-TT***	**m.15928G>A**	–	**Substitution**	**Homoplasmy**
	*MT-ND2*	m.5231G>A	p.Leu254(=)	Silent	Homoplasmy		*MT-ATP6*	m.8697G>A	p.Met57(=)	Silent	Homoplasmy
	*MT-CO1*	m.6185T>C	p.Phe94(=)	Silent	Homoplasmy		*MT-ND5*	m.12633C>T	p.Ser99(=)	Silent	Homoplasmy
	*MT-CO1*	m.7146A>G	p.Thr415Ala	Missense	Homoplasmy		*MT-ND5*	m.13368G>A	p.Gly344(=)	Silent	Homoplasmy
	*MT-ATP8*	m.8428C>T	p.Phe21(=)	Silent	Homoplasmy		*MT-CYB*	m.14905G>A	p.Met53(=)	Silent	Homoplasmy
	*MT-ATP8*	m.8468C>T	p.Leu35(=)	Silent	Homoplasmy		*MT-CYB*	m.15607A>G	p.Lys287(=)	Silent	Homoplasmy
	*MT-ATP6*	m.8566A>G	p.Ile14Val	Missense	Homoplasmy	P18	D-loop	m.16234C>T	–	Substitution	Homoplasmy
	*MT-ATP6*	m.8655C>T	p.Ile43(=)	Silent	Homoplasmy		D-loop	m.16257C>T	–	Substitution	Homoplasmy
	*MT-ATP6*	m.9042C>T	p.His172(=)	Silent	Homoplasmy		D-loop	m.16259C>T	–	Substitution	Homoplasmy
	*MT-CO3*	m.9288A>G	p.Thr28Ala	Missense	Homoplasmy		D-loop	m.16269A>G	–	Substitution	Homoplasmy
	*MT-CO3*	m.9347A>G	p.Leu47(=)	Silent	Homoplasmy		D-loop	m.16290C>T	–	Substitution	Homoplasmy
	*MT-CO3*	m.9755G>A	p.Glu183(=)	Silent	Homoplasmy		*MT-RNR2*	m.1927G>A	–	Substitution	Heteroplasmy
	*MT-CO3*	m.9818C>T	p.His204(=)	Silent	Homoplasmy		*MT-RNR2*	m.2283C>T	–	Substitution	Heteroplasmy
	*MT-ND4L*	m.10664C>T	p.Val65(=)	Silent	Homoplasmy		*MT-CO1*	m.6546C>T	p.Leu215Phe	Missense	Homoplasmy
	*MT-ND4L*	m.10688G>A	p.Val73(=)	Silent	Homoplasmy		*MT-CO1*	m.6599A>G	p.Gln232(=)	Silent	Homoplasmy
	***MT-ND4***	**m.10810T>C**	**p.Leu17(=)**	**Silent**	**Homoplasmy**		*MT-CO2*	m.7681C>T	p.Phe32(=)	Silent	homoplasmy
	***MT-ND4***	**m.10915T>C**	**p.Cys52(=)**	**Silent**	**Homoplasmy**		*MT-CO2*	m.7762G>A	p.Gln59(=)	Silent	Homoplasmy
	***MT-ND4***	**m.11176G>A**	**p.Gln139(=)**	**Silent**	**Homoplasmy**		*MT-ND5*	m.12771G>A	p.Glu145(=)	Silent	Homoplasmy
	*MT-ND4*	m.11641A>G	p.Met294(=)	Silent	Homoplasmy		*MT-ND5*	m.13588C>T	p.Leu418(=)	Silent	Homoplasmy
	***MT-ND4***	**m.12007G>A**	**p.Trp416(=)**	**Silent**	**Homoplasmy**		*MT-CYB*	m.15884G>C	p.Ala380Pro	Missense	Heteroplasmy
	*MT-ND4*	m.12031G>A	p.Asn424Lys	Missense	Homoplasmy	P22	D-loop	m.58T>C	–	Substitution	Heteroplasmy
	*MT-ND5*	m.13276A>G	p.Met314Val	Missense	Homoplasmy		D-loop	m.196T>C	–	Substitution	Homoplasmy
	*MT-ND5*	m.13506C>T	p.Tyr390(=)	Silent	Homoplasmy		*MT-RNR1*	m.827A>G	–	Substitution	Heteroplasmy
	*MT-ND6*	m.14308T>C	p.Gly122(=)	Silent	Homoplasmy		*MT-RNR2*	m.3184C>T	–	Substitution	Homoplasmy
	*MT-ND6*	m.14569G>A	p.Ser35(=)	Silent	Homoplasmy		*MT-ND2*	m.5191C>T	p.Thr241Met	Missense	Homoplasmy
	*MT-CYB*	m.15136C>T	p.Gly130(=)	Silent	Homoplasmy		*MT-CO3*	m.9419C>T	p.His71(=)	Silent	Homoplasy
	*MT-CYB*	m.15431G>A	p.Ala229Thr	Missense	Homoplasmy		*MT-ND3*	m.10373G>A	p.Glu105(=)	Silent	Homoplasmy
							*MT-ND4*	m.11761C>T	p.Tyr334(=)	Silent	Homoplasmy
							*MT-CYB*	m.15850T>G	p.Thr368(=)	Silent	Homoplasmy

*LHON* Leber’s hereditary optic neuropathy, *RRMS* relapse-remitting multiple sclerosis. Mutations in bold were previously reported in LHON patients according to MITOMAP, HmtDB, ClinVar and MEDLINE-listed publications on life sciences.

### LHON-related mtDNA variants in RRMS patients and healthy controls

We also investigated the presence of mtDNA variants in RRMS patients and healthy controls. The results (Table 5) revealed a number of mtDNA SNVs in protein-coding genes, all were in the homoplasmic state and were previously reported in LHON patients. Comparison of the frequency of individual variant between patients and controls showed statistically significant differences in the prevalence of five variants (P < 0.05). The association of these variants with MS was confirmed by the Fisher's exact test and Bonferroni correction test.

**Table 5:** LHON-related mtDNA mutations in RRMS patients and healthy controls.

Gene	Nucleotide change	Amino acid change	Type of mutation	Homoplasmy/heteroplasmy	Mutation ID	Frequency (%)		OR	95% CI	P value (Fisher's exact test)	P value (Bonferroni correction)	Interpretations of pathogenicity*
						Patients	Controls					
*MT-ND1*	m.4216T>C	p.Tyr304His	Missense	Homoplasmy	rs1599988	39	13	3.13	0.97–10.1	0.049	0.037	Conflicting interpretations of pathogenicity
*MT-CO1*	m.7028C>T	p.Ala375(=)	Silent	Homoplasmy	rs2015062	96	42	2.30	1.42–3.72	< 0.01	< 0.01	NP
*MT-ND3*	m.10398A>G	p.Thr114Ala	Missense	Homoplasmy	rs2853826	30	63	0.49	0.24–0.97	0.041	0.028	Benign/protective
*MT-ND5*	m.13708G>A	p.Ala458Thr	Missense	Homoplasmy	rs28359178	39	8	4.69	1.13–19.5	0.017	0.012	Conflicting interpretations of pathogenicity
*MT-CYB*	m.14766C>T	p.Thr7Ile	Missense	Homoplasmy	rs193302980	96	50	1.91	1.27–2.88	< 0.01	< 0.01	Benign

*LHON* Leber’s hereditary optic neuropathy, *RRMS* relapse-remitting multiple sclerosis, *OR* odds ratio, *CI* confidence interval, *NP* not provided. *Interpretation was reported according to ClinVar database.

Short reads mapped against mitochondrial genome of these variants are shown in Supplementary S1 Fig, S2 Fig, S3 Fig, S4 Fig and S5 Fig. Among them, three missense variants and one silent variant were more prevalent in the patient group: The missense variant m.4216T>C (referred as rs1599988 polymorphism) in *MT-ND1* gene occurred in 39% of patients vs 13% of controls (OR 3.13, 95% CI 0.97–10.1). The missense variant m.13708G>A (refereed as rs28359178 polymorphism) in *MT-ND5* gene occurred in 39% of patients vs 8% of controls (OR 4.69, 95% CI 1.13–19.5). The missense variant m.14766C>T (referred as rs193302980 polymorphism) in *MT-CYB* gene occurred in 96% of patients vs 50% of controls

(OR 1.91, 95% CI 1.27–2.88). The silent variant m.7028C>T (referred as rs2015062 polymorphism) in *MT-CO1* gene occurred in 96% of patients vs 42% of controls (OR 2.30, 95% CI 1.42–3.72). However, according to ClinVar database, the variants m.4216T>C and m.13708G>A showed conflicting interpretations of pathogenicity, whereas m.14766C>T was classified as a benign variant. Only one missense variant namely m.10398A>G (referred as rs2853826 polymorphism) in *MT-ND3* gene occurred less frequently in patients (30%) compared to controls (63%) (OR 0.49, 95% CI 0.24–0.97). This variant was classified as having a benign/protective effect according to ClinVar database.

Other variants showed no statistical significant differences in their prevalence among patients and controls, and represent simple polymorphisms with no pathological impact, thus they were not analyzed further.

## Discussion

MS has long been known to be associated with LHON, a mitochondrial inherited form of vision loss caused by point mutations in mtDNA genes. Both primary and secondary LHON variants have been reported in MS^17,25–30^. However, the presence and relative frequencies of LHON variants in MS patients vary between populations, possibly due to genetic, racial and other factors, which differ in different ethnicities^25–33^. In the current study, we analyzed our recently generated large-scale genomic sequence data of the entire mtDNA from 47 unrelated Saudi Arab individuals, 23 patients diagnosed with RRMS and 24 healthy control subjects to investigate the presence of mutations/variants involved in or associated with LHON in those subjects. Our mutational analysis revealed ten LHON-related variants exclusively present in RRMS patients and not in healthy subjects (Table 2). Three of them were pathogenic/provisional pathogenic mutations and detected in two patients. The homoplasmic m.14484T>C mutation was found in patient P2. m.14484T>C mutation which causes an amino acid change from methionine to valine at position 64 (Met64Va) within the *MT-ND6* gene is one of the well-known primary LHON-causing mutation and was also described in a young man with an MS-like illness^26^. While this mutation infrequently found in LHON patients in most countries^17,38^, it is the most common mutation detected among French-Canadians due to a founder effect^39^. The heteroplasmic m.10237T>C mutation was also found in patient P2. m.10237T>C mutation which causes an amino acid change from isoleucine to threonine at position 60 (Ile60Thr) within the *MT-ND3* gene is a rare LHON mutation, reported in two Hungarian siblings with LHON patients lacking the three most common pathogenic DNA mutations^40^. It was also described in eleven patients with LHON of European ancestry harbouring the primary mutation m.14484T>C^41^. Based on predictive analysis of the impact on protein function (Table 2), both mutations were classified as deleterious, having probably damaging/damaging effects by the PolyPhen2, SIFT, CAAD, and Mutation Assessor models. In our previous study^34^, we also showed that these two mutations affect the structures of encoded-proteins. The homoplasmic m.9101T>C mutation was found in another patient (P15). This mutation which causes an amino acid change from isoleucine to threonine at position 192 (Ile192Thr) within the *MT-ATP6* gene is a rare LHON mutation reported in a single affected patient^42^. Although the site of this mutation is not in the conserved region of the *MT-ATP6* gene, it was linked with decreased coupling of proton flow with ATP production^42^, but without inhibition of respiration^43^. Highly conserved residues among homologous proteins are generally considered to be critical for protein stability, interaction and function. In our bioinformatics analysis, the effect of this mutation on encoded-protein was predicted to be benign, neutral and low by the PolyPhen2 SIFT, CAAD and Mutation Assessor models respectively (Table 2).

In addition, seven secondary LHON-related SNVs, all in the homoplasmic state, were also detected in the patient group (Table 2). Of them, three were found in two patients along with the primary/rare LHON mutations. These include m.5442T>C in *MT-ND2* gene and m.13105A>G in *MT-ND5* which were detected in patient P2 and m.12358A>G in *MT-ND5* which was found in patient P15. The other four variants namely m.4695T>C in *MT-ND2* gene, m.4917A>G in *MT-ND2* gene, m.3316G>A in *MT-ND1* gene and 3533C>T in *MT-ND1* were individually found in different other patients (P1, P16, P18 and P22 respectively). In previous studies, these secondary variants were described in LHON or LHON-like cases from different ethnic backgrounds along with other LHON primary mutations, and suggested to modify the effect of primary mutations^44–46^. Interestingly, m.4659G>A was also reported as a benign variant associated with Parkinson’s disease, a progressive neurodegenerative disorder^47^. Our prediction analysis revealed that all these secondary variants have benign, neutral and low effects by the PolyPhen2 SIFT, CAAD and Mutation Assessor models respectively (Table 2).

In our study, most of the identified LHON mutations/variants in RRMS patients were located in NADH dehydrogenase subunit genes of complex I, except for one rare mutation in *MT-ATP6* gene of complex V. Mutations in mtDNA-encoded genes of the mitochondrial electron transport chain complexes can lead to deficient function of OXPHOS, decrease in ATP synthesis, and increase in ROS production^11,48^. Importantly, defects in mitochondrial OXPHOS mainly affect tissues with a high energy demand such as brain, nerves, retina, skeletal and cardiac muscle. While earlier reports showed the co-occurrence of LHON mutations in a sub-group of MS patients or patients with MS-like illness^17,25–28^, the association of primary LHON mutations with MS was suggested to be more than a coincidence and individuals carrying primary LHON mutations are at risk of developing MS^29^. There is an overlap in the clinical and molecular features of MS and LHON. At the molecular level, the interaction between mtDNA mutations in LHON and MS has been suggested as converge on shared pathways of oxidative damage and cell death^49^. Particularly, complex I respiratory chain dysfunction caused by LHON mtDNA mutations has been shown to decrease energy production and increase ROS generation, leading to retinal ganglion cell defect and apoptosis^48,49^. These impairments are also seen in demyelination and neurodegeneration in MS whereby complex I dysfunction leads to mitochondrial dysfunction and energy failure^7–9^. Moreover, mtDNA mutations underlying LHON may contribute to presumably immunologically mediate involvement of other myelinated axons in the CNS in susceptible individuals^28^. The presence of primary/rare pathogenic LHON mutations in some patients with RRMS in this study may suggests a possible role for these mutation in the pathogenesis of MS. Secondary LHON-associated variants may have synergistic effects with the primary mutations or may be responsible for disease phenotype difference^22–24^. Therefore, secondary LHON variants with uncertain pathological significance may also play a role in MS. Further investigations are fundamental to verify the link between LHON-related variants and MS, because the etiology of MS is still unknown.

All patients reported here were first diagnosed as being affected with MS and had both clinical and MRI features of RRMS based on the McDonald diagnosis criteria^35^. Five out of the six patients who harboured LHON variants were females (P1, P15, P16, P18, P22) and one was a male patient (P2) (Table 3). They were all young adults with different disease durations and different disability scales as indicated by their EDSS. Along with other neurological dysfunctions such as weakness and balance problem, the patients were reported with numbness and visual problem. Visual problem is one of the most common symptoms of MS caused by inflammation in various parts of the optic nerve (optic neuritis) and the pathways from the brain to the eyes and eye muscles^2^. Unfortunately, we do not know if the family members of our patients are affected by LHON, as genetic testing for LHON is not routinely performed when the initial diagnosis of MS was considered. While there is no evidence to support screening for LHON mutations in all MS patients^20^, mtDNA analysis is appropriate in a sub-group of MS patients who harbour pathogenic LHON mutations^27^. Our observations of the presence of pathogenic/provisional pathogenic LHON mutations in two RRMS patients (P2 and P15) highlight the importance of performing molecular genetic analysis not only for patients, but for all relatives in the maternal line to seek guidance on whether they have affected family members and offspring. Molecular genetic testing can be also offered to exclude the possibility of de novo LHON mutations. De novo mutations in LHON are extremely rare, but have been reported for m.14484T>C mutation and other mutations^18,19^.

Although the penetrance of LHON pedigrees is determined primarily by a mutation in the mtDNA, other factors such as mitochondrial genetic background, nuclear genetic factors and environmental factors are also necessary for manifestation and severity of the disease^18–21^. Since our patients were initially diagnosed with MS, many factors need to be addressed to determine the penetrance of the pathogenic/rare mutations. Therefore, further studies are required to investigate the family members of the patients to determine the number of affected males to females, and to assess if they do or not develop features of LHON. Moreover haplogroup analysis is also important for the clustering of theses mutations in the Arab ethnic group. For instance, LHON m.11778G>A and m14484T>C mutations are clustered in haplogroup J in European populations, whereas these two mutations are not associated with haplogroups J but in the Asian haplogroups M and BM, suggesting that different sets of SNPs from the European LHON-related haplotype may contribute to the Asian LHON onset^50^.

When we investigated the presence of other mtDNA variants in the RRMS patients who carried one of the LHON-related variants, we found that these patients exhibited distinct sets of mtDNA SNVs in different regions of the mitochondrial genome (Table 4). Of them, eight were previously reported in patients with LHON. In the D-loop region, the variants m.189A>G and m.236T>C were found in one patient (P2). Both variants were previously reported in Chinese patients carrying other LHON mutations^51^. The D-loop is the non-coding region in mtDNA and contains essential transcription and replication elements, thus contributes to the proper functioning of mitochondria^52^. Variants, which are more frequently occur in the D-Loop region have been implicated in several diseases including neurological disorders^53^. Another variant namely m.15928G>A in *MT-TT* gene-encoding tRNA^Thr^ was found in one patient (P16). This variant was also described in Chinese subjects with LHON and was suggested to influence the phenotypic manifestation of LHON by affecting the function of *MT-TT* gene, leading to impairment of mitochondrial protein synthesis and deficient respiration^54^. Five more SNVs represented silent variants were found in *MT-ND4* gene. Four of them namely m.10810T>C, m.10915T>C, m.11176G>A, and m.12007G>A were detected in patient P2 and m.10876A>G was found in patient P15. All of these silent variants were previously described in Chinese subjects carrying LHON mutations^51,54^. Although silent substitutions do not alter the amino acids and hence the protein sequence, they are suspected to have a potential involvement in human diseases by altering transcript splicing, mRNA stability, or even protein structure and function^55,56^. The presence of numerous mtDNA variants in RRMS patients who possess one of the LHON mutations might modify the effect of these mutations. The functional significances of these variants should be further examined, especially in patient P2 who was noticed to carry most of these variants along with the pathogenic/rare LHON mutations and secondary variants.

Our analysis of the entire mtDNA in patients and controls revealed a number of variants in protein-coding genes, all were in the homoplasmic state (Table 5) and were previously described as secondary LHON-related variants^22,57,58^. Five of these variants differed significantly in their prevalence among patients and controls (P < 0.05). Specifically, three missense variants and one silent variant were more prevalent in patients than in controls.

The missense variants include: m.4216T>C referred as rs1599988 polymorphism in *MT-ND1* gene was observed in 39% of patients vs 13% of controls (OR 3.13, 95% CI 0.97–10.1), m.13708G>A refereed as rs28359178 polymorphism in *MT-ND5* gene was observed in 39% of patients vs 8% of controls (OR 4.69, 95% CI 1.13–19.5), m.14766C>T referred as rs193302980 polymorphism in *MT-CYB* gene was found in 96% of patients vs 50% of controls (OR 1.91, 95% CI 1.27–2.88). One silent variant namely m.7028C>T referred as rs2015062 polymorphism in *MT-CO1* gene was found in 96% of patients vs 42% of controls (OR 2.30, 95% CI 1.42–3.72). These variants were previously described in MS cases and healthy controls^14,33,59^. Particularly, m.4216T>C and m.13708G>A variants were considered as predisposing markers to MS^33,59^. Both of them were also suggested as contributing factors of optic neuritis in MS patients^60,61^. According to ClinVar database, the variants m.4216T>C and m.13708G>A showed conflicting interpretations of pathogenicity, whereas m.14766C>T was classified as a benign variant. Only one missense variant namely m.10398A>G referred as rs2853826 polymorphism in *MT-ND3* gene was found less frequently in patients (30%) compared to controls (63%) (OR 0.49, 95% CI 0.24–0.97), suggesting a protective effect of this variant in MS. This variant was classified as having a benign/protective effect according to ClinVar database. It was found to be associated with the reduced risk of Parkinson disease, and its protective effect was proposed to increase the performance of complex I within the brain and other tissues in individuals belong to specific haplogroups^62^. A protective effect of mtDNA variants has been reported for several diseases such as for haplogroup M with reduced the risk of visual failure in European families with m.11778G>A LHON mutation^58^. Even though these results indicate that particular mtDNA SNVs differ in their prevalence among MS patients and controls, further validations are required to conclude their exact association with the risk of MS.

Results from the present study, the first to investigate LHON mtDNA mutations/variants in Saudi patients with RRMS and healthy controls, suggest a possible role of the primary/rare LHON mutations in the pathogenesis of MS and a potential association of secondary LHON-related variants with the genetic predisposing to MS. While our study provide important information on the occurrence and association of LHON mtDNA mutations/variants with MS in an Arab population, it has some limitations. The sample size was relatively small and further studies are needed in a large-scale. Moreover, genetic screening of a selected MS patients for pathogenic LHON mutations, pedigree analysis, and additional functional and structural investigations of LHON-related variants are required to provide a better insight into the involvement of these variants in MS.

## Methods

### Study subjects

The study included 47 unrelated Saudi Arab individuals, 23 patients with RRMS and 24 healthy controls. Patients were recruited from the Neurology Clinic at King Khalid Hospital, King Saud University, Saudi Arabia. Patients were classified as RRMS when they satisfied McDonald diagnosis criteria^35^, with at least two previous relapses in the central nervous system regions, confirmed by a neurological examination, magnetic resonance imaging (MRI) scans, and electrophysiological studies. Control subjects without neurological conditions or history of autoimmune and inflammatory disease were King Khalid hospital blood donors. Demographic data (Age at the onset of the first symptoms and gender) and clinical data (disease duration, disability status evaluated using the Kurtzke Expanded Disability Status Scale [EDSS], clinical features and medications) were reported for all patients. Other data including blood pressure and body mass index (BMI) were also recorded for both patients and controls. Written informed consents were obtained from all participants. The study was approved by the Scientific and Ethics Committee in King Saud University, College of Medicine (Saudi Arabia), and the Medical Research and Ethics Committee in the College of Medicine and Medical Sciences, Arabian Gulf University (Bahrain). All methods were performed in accordance with the relevant guidelines and regulations.

### DNA extraction, next generation sequencing and analysis

DNA was extracted in our previous study^34^ from the blood of RRMS patients and healthy controls using the QIAMP DSP DNA kit (Qiagen, Hilden, Germany), and all extracted DNA samples were quantified and checked for purity using the NanoDrop ND-1000 ultraviolet–Visible light spectrophotometer (Thermo Fisher Scientific, Inc.). High-throughput sequencing of mitochondrial genomes was performed as described previously^34^. In brief, whole mtDNA was amplified using long-range PCR kit (Qiagen) PCR products were subsequently purified and quantified before proceeding to DNA library preparation for next-generation sequencing (NGS) using Illumina protocol and sequencing on HiSeq X instrument. For sequence analysis, paired end sequencing data were exported to FASTQ file. Then the sequence reads were trimmed using custom script to remove adapters and bases with low quality value. Alignment of the trimmed reads was performed to hg19 version of the genome available from UCSC genome browser. A sequencing coverage of 10,000 X of the mitochondrial genome was achieved, at such coverage heteroplasmy at 5% levels would be detectable. After alignment, the mtDNA variants were compared with the Revised Cambridge Reference Sequence (rCRS), (NCBI Reference Sequence: NC_012920). mtDNA variants were annotated using the MITOMAP database system for the human mitochondrial genome (http://www.mitomap.org/MITOMAP) and other databases. In general, mtDNA variants can be classified into three categories^36^:

1. Benign variant: If a variant has been reported in MITOMAP as a polymorphism, has not been associated with a disease in the population or family studies, and has been reported in mtDB at a frequency > 0.2%.
2. Unclassified variant: A novel variant, a rare variant that has been reported in MITOMAP as a polymorphism, or reported in mtDB at a frequency ≤ 0.2%, and a rare variant reported in the literature or MITOMAP as a mutation based on a single family study/single report with no functional studies for their pathogenicity. These variants must be further evaluated by protein structure prediction.
3. Mutation: a variant that has been listed in MITOMAP as confirmed mutation and has been reported in multiple unrelated patients/families with clinical correlation and or supporting functional prediction.

For the identification of LHON mutations/variants, extensive search was done through several databases including MITOMAP, HmtDB (Human Mitochondrial Genome Database), ClinVar and MEDLINE-listed publications on life sciences.

### Bioinformatics analysis

We used four bioinformatics tools (PolyPhen2, SIFT, CADD and Mutation Assessor) to determine the deleteriousness of nonsynonymous mutations. These tools predict the impact of nonsynonymous mutations on protein function and structure based on sequence homology, evolutionary conservation, and protein structural information^37^. The prediction of PolyPhen (Polymorphism Phenotyping) is provided as benign, possibly damaging, and probably damaging with scores of < 0.446, > 0.446, and > 0.908 respectively. The prediction of SIFT (Sorting Intolerant From Tolerant) is provided as tolerated with a score of ≥ 0.05, whereas a score of < 0.05 is considered as damaging. The prediction of CADD (Combined Annotation Dependent Depletion) is provided as benign and deleterious with scores range from 1 to 99 (higher scores indicate more deleterious variants). The Mutation Assessor prediction is provided as neutral, low, medium, or high with scores ranging from 0 to 1 (higher scores indicate more deleterious variants).

### Statistical analysis

Comparisons between patients and controls were evaluated using a Chi-square test for categorical variables or equivalent non-parametric Wilcoxon signed-rank test and Mann–Whitney test for normally distributed variables. Differences in the frequencies of variants among cases and controls were assessed using the Fischer exact test. The association of variants with MS was done using the Fisher's exact test, and the odds ratios (OD) and 95% confidence interval (95% CI) were reported. Moreover, multiple comparison correction was also done using the Bonferroni correction. A P value of < 0.05 was considered as statistically significant. All data were analysed using the SPSS software (version 23; IBM Corp., Armonk, NY, USA).

## Supplementary Information

Supplementary Figure S1.

Supplementary Figure S2.

Supplementary Figure S3.

Supplementary Figure S4.

Supplementary Figure S5.

## Data Availability

The data sets of the entire mtDNA sequences were previously registered in the Sequence Read Archive Repository (ref. No. PRJNA781092)^34^.
