# Supplementary figures and images for: Analysis of the entire mitochondrial genome reveals Leber’s hereditary optic neuropathy mitochondrial DNA mutations in an Arab cohort with multiple sclerosis
Source: Sci Rep. 2022 Jun 30;12:11099. doi: 10.1038/s41598-022-15385-2 (PMC9246974; doi:10.1038/s41598-022-15385-2)

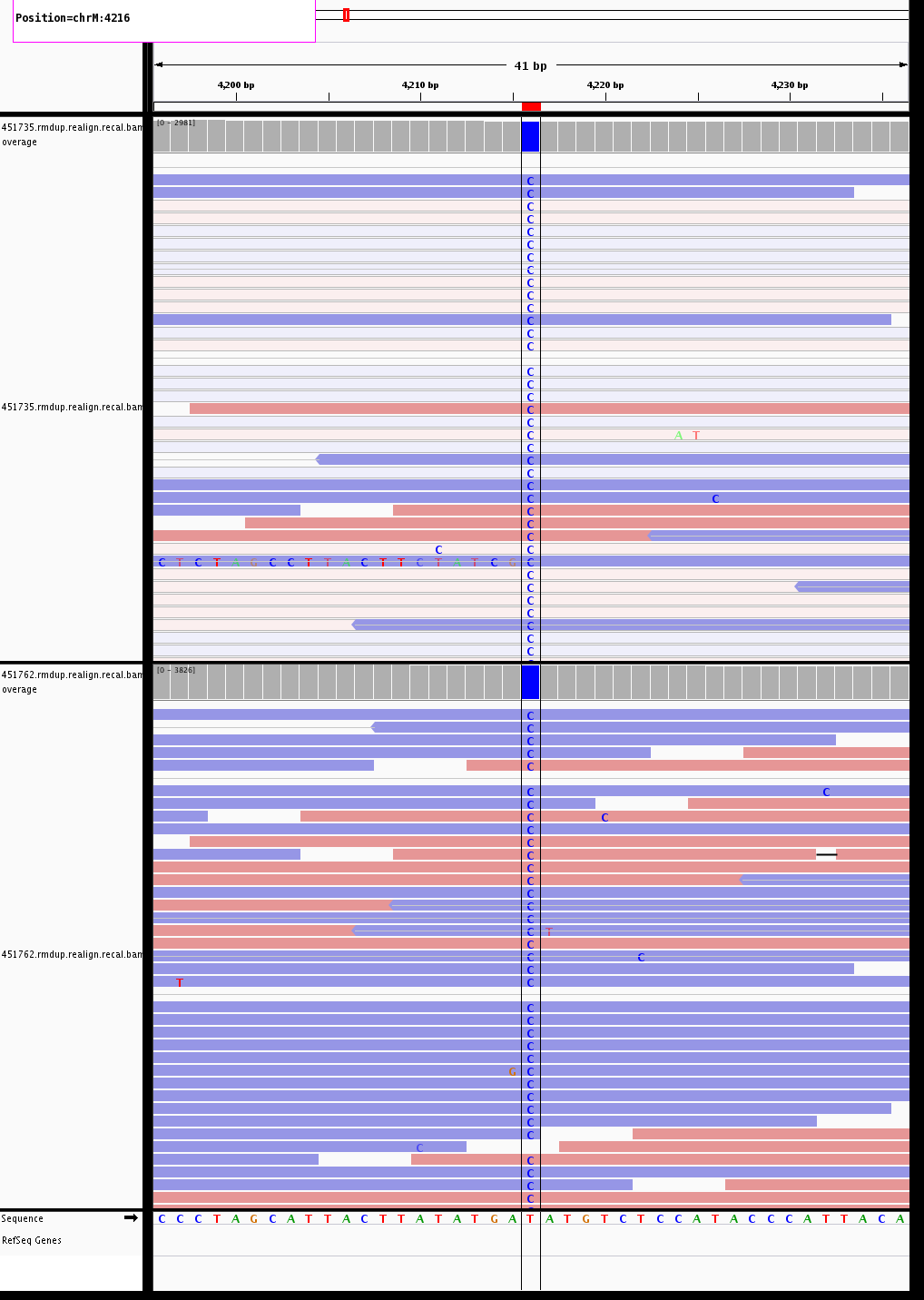


S1 Fig. HiSeq X NGS short reads of the secondary LHON-related variant m.4216T>C (p.Tyr304His) of *MT-ND1* gene.

Supplement: Supplementary file 1 — Supplementary Figure S1. [file 41598_2022_15385_MOESM1_ESM.docx]

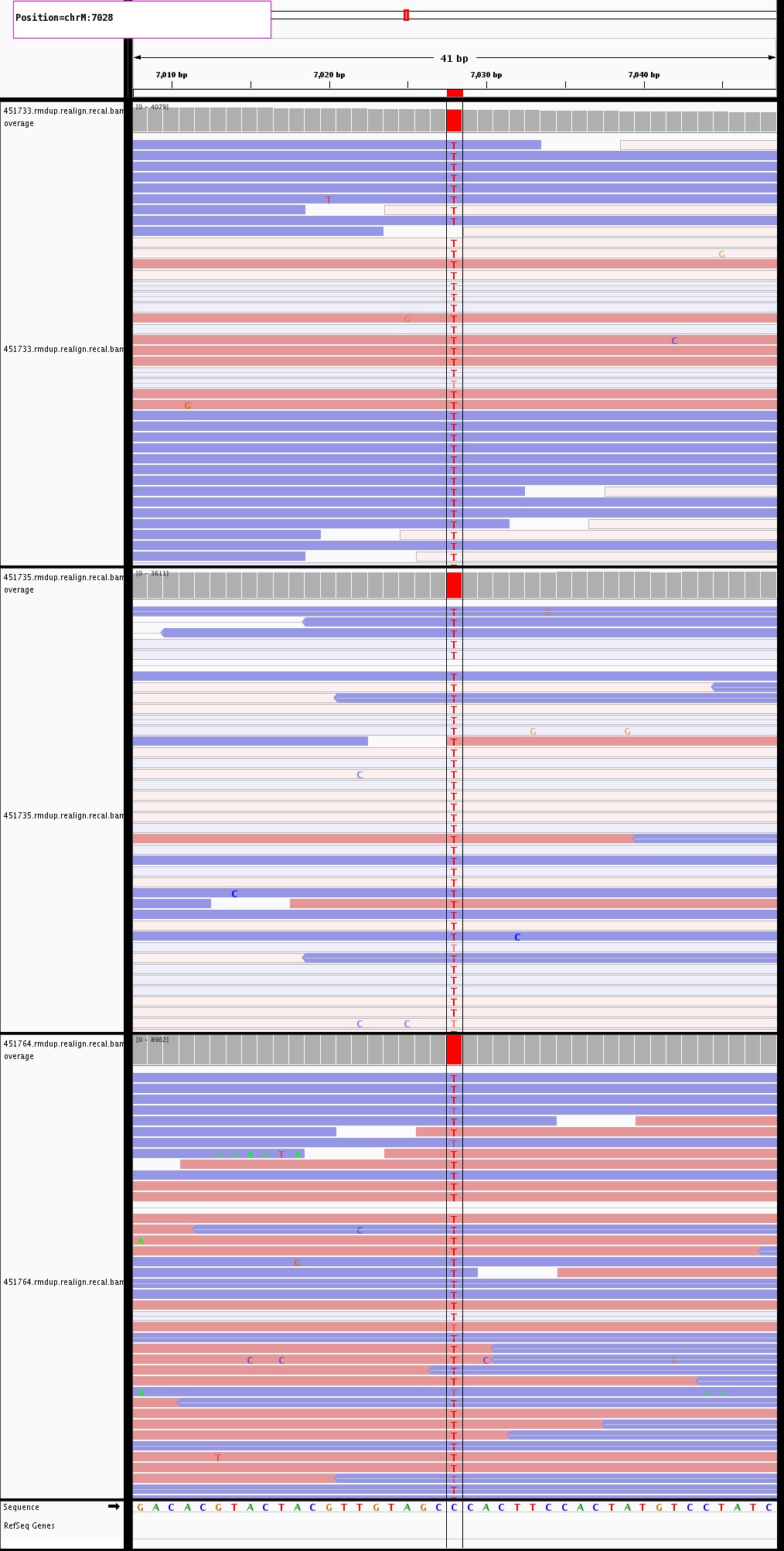


S2 Fig. HiSeq X NGS short reads of the secondary LHON-related variant m.7028C>T (p.Ala375(=)of *MT-CO1* gene.

Supplement: Supplementary file 2 — Supplementary Figure S2. [file 41598_2022_15385_MOESM2_ESM.docx]

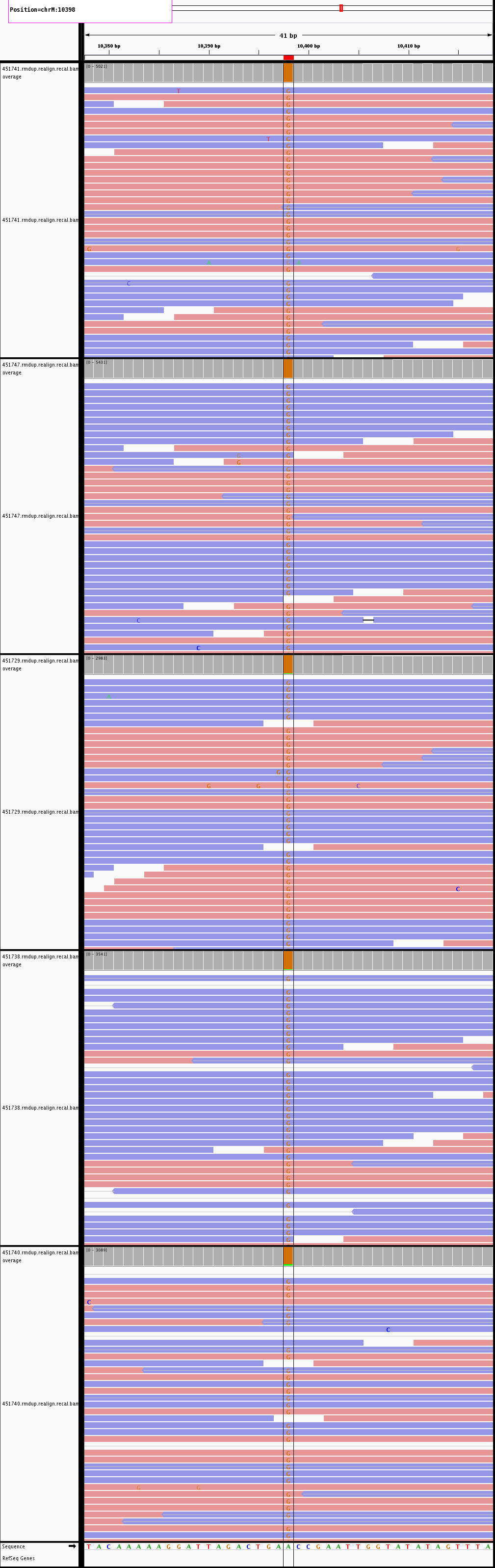


S3 Fig. HiSeq X NGS short reads of the secondary LHON-related variant m.10398A>G (p.Thr114Ala) of *MT-ND3* gene.

Supplement: Supplementary file 3 — Supplementary Figure S3. [file 41598_2022_15385_MOESM3_ESM.docx]

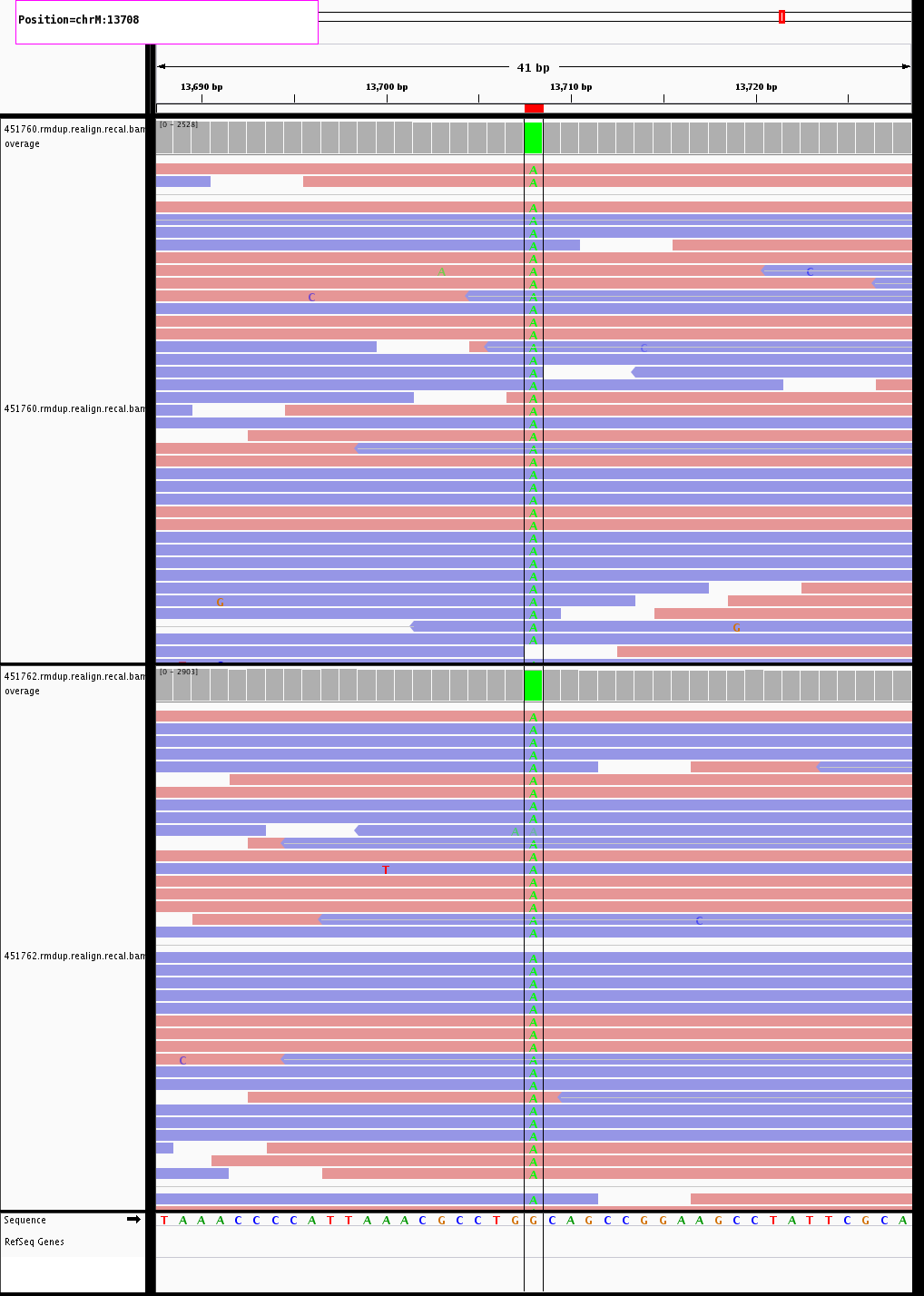


S4 Fig. HiSeq X NGS short reads of the secondary LHON-related variant m.13708G>A (p.Ala458Thr) of *MT-ND5* gene.

Supplement: Supplementary file 4 — Supplementary Figure S4. [file 41598_2022_15385_MOESM4_ESM.docx]
