# Supplementary material for: Analysis of the entire mitochondrial genome reveals Leber’s hereditary optic neuropathy mitochondrial DNA mutations in an Arab cohort with multiple sclerosis
Source: Sci Rep. 2022 Jun 30;12:11099. doi: 10.1038/s41598-022-15385-2 (PMC9246974; doi:10.1038/s41598-022-15385-2)
Supplement: Supplementary file 5 — Supplementary Figure S5. [file 41598_2022_15385_MOESM5_ESM.docx]

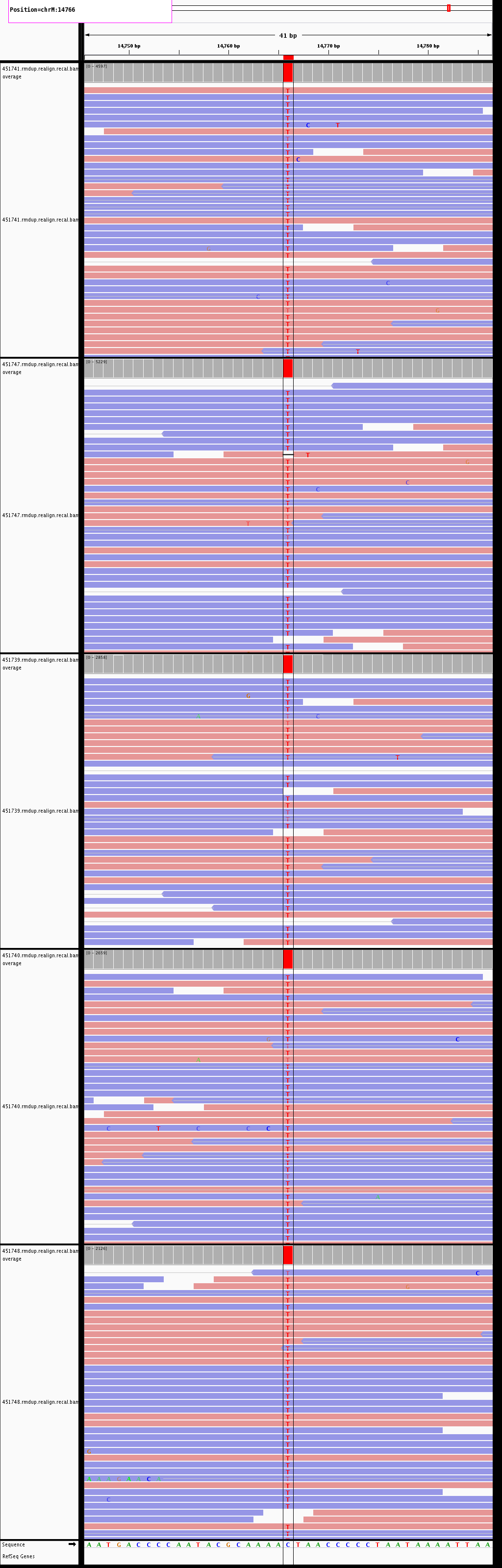


S5 Fig. HiSeq X NGS short reads of the secondary LHON-related variant m.14766C>T (p.Thr7Ile) mutation of *MT-CYB* gene.
